# Supplementary material for: Implementation of a Conditional Latent Diffusion-Based Generative Model to Synthetically Create Unlabeled Histopathological Images
Source: Bioengineering (Basel). 2025 Jul 15;12(7):764. doi: 10.3390/bioengineering12070764 (PMC12292637; doi:10.3390/bioengineering12070764)
Supplement: Supplementary file 1 [file bioengineering-12-00764-s001.zip › bioengineering-3724138-supplementary.pdf]

Supplementary Information

1. conditional Latent Diffusion Model (cLDM) Architecture

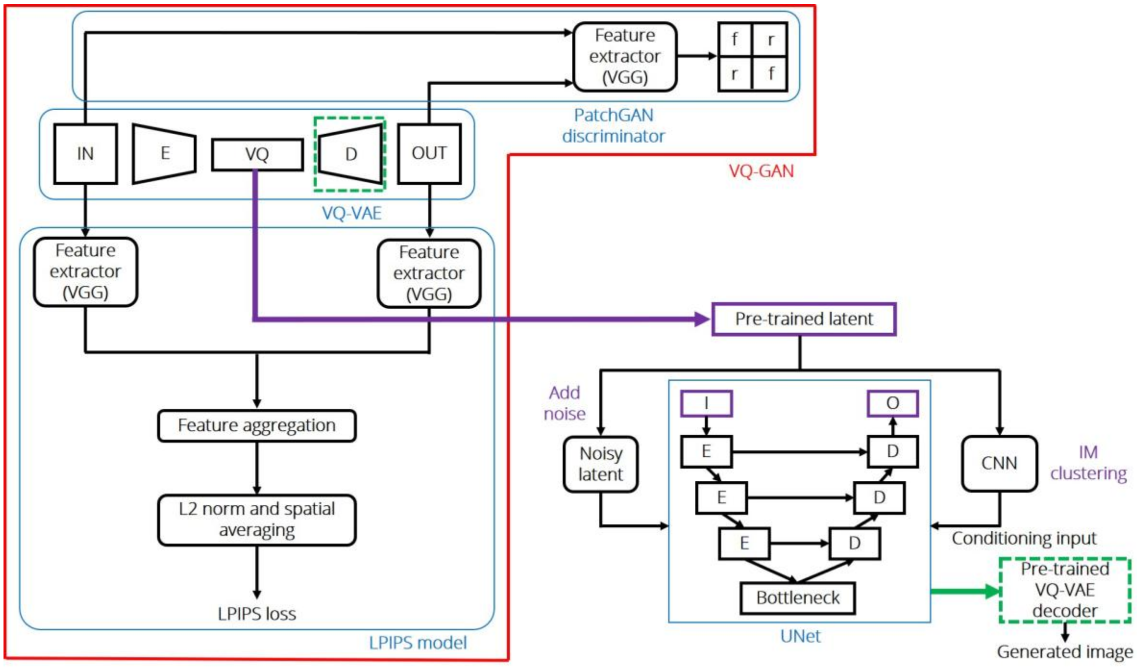

Figure S1: The architecture of our proposed conditional latent diffusion model (cLDM).

Figure S1 presents our proposed cLDM architecture. The autoencoder model (VQ-GAN) is composed of three components: the VQ-VAE, the LPIPS model, and the PatchGAN discriminator. We first train the VQ-GAN and then use the pre-trained latent space for the diffusion process. The UNet is responsible for recovering the clean latent representation from the noisy latent, which is then passed to the pre-trained VQ-VAE decoder to generate samples. A neural network (CNN) is used to perform clustering on the pre-trained latent features, and the resulting clusters serve as a conditioning mechanism for the UNet to generate specific samples.

2. cLDM Loss Function

Equation S1 presents the loss function (L_F) for our cLDM architecture.

$$L_F = L_{MSE} - \big(\lambda_{ME} \times H(Y) \; - \; \lambda_{CE} \times H(Y|X)\big) + \lambda_{AF} \times L_{AF} \qquad \text{(S1)}$$

Here, L_{MSE} represents the error between the actual and predicted noise. The second term denotes information maximization, defined as the difference between marginal entropy and conditional entropy; a higher value indicates better clustering. $H(Y)$ and $H(Y|X)$ represent the marginal and conditional entropy, respectively. L_{AF} denotes the affine loss, which arises from applying various transformations. The coefficients λ_{ME}, λ_{CE}, and λ_{AF} serve as trade-off parameters for marginal entropy, conditional entropy, and affine loss, respectively.

3. Variance Scheduler

We used a linear variance scheduler in our work. In diffusion models, the variance scheduler controls the amount of noise added at each timestep.

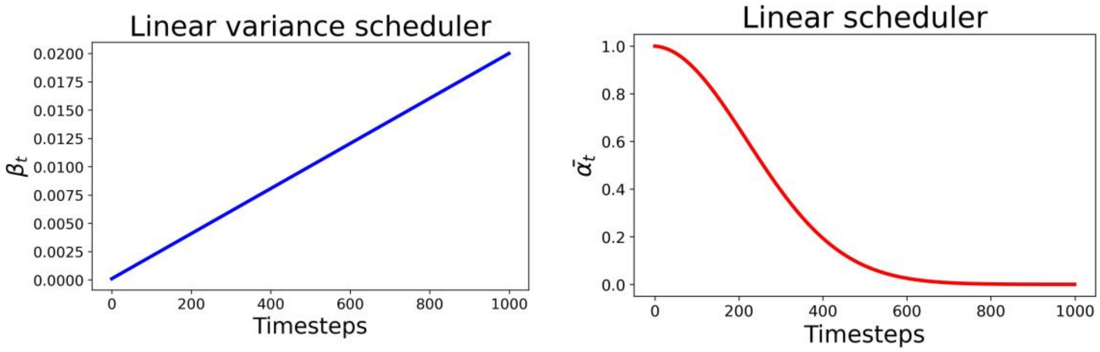

(a) (b)

Figure S2: β_t and $\bar{\alpha}_t$ plots for linear variance scheduler.

Figure S2 shows the plots of β_t and $\bar{\alpha}_t$ for the linear variance scheduler. The β_t plot illustrates how the diffusion process progresses over a finite number of timesteps. For our research, we selected 1000 timesteps, with β_t values starting at 0.0001 for the first timestep and increasing linearly to 0.02 at the last timestep, as shown in Figure 2(a). The $\bar{\alpha}_t$ plot indicates how quickly or slowly the information in the source image is lost during the diffusion process. When $\bar{\alpha}_t$ reaches zero, as seen in Figure 2(b), the information in the source image is considered to be completely destroyed.
